# The Origin and Evolution of the 18th Century Hospital Movement
*Previous articles appeared on December 13, 1913, January 17 and 31, and February 14, 1914.


**Published:** 1914-02-28

**Authors:** 


### V. THE ORIGIN AND EVOLUTION OF THE 18th CENTURY HOSPITAL MOVEMENT.*

In 1721 Thomas Guy, who had already been a munificent benefactor to St. Thomas's, announced his intention of erecting and endowing a hospital for incurables, thereby "establishing an asylum for that stage of languor and disease to which the charity of others had not reached, providing a retreat for hopeless insanity, and rivalling the endowment of kings." But although it was primarily intended for the reception of those poor persons who, though capable of relief by physic or surgery, by reason of the small hopes there might be of their cure or the length of time necessary for that purpose, might be judged incurable by other hospitals, his executors and trustees were empowered, if they should think fit, to fill all or a great part of their beds with other patients.†

Considering the enormous amount of Guy's benefaction, representing more than a million of money according to present value, his foundation was beyond the need of outside subscription; and whereas, bearing in mind the late numerous instances of the maladministration of private charities, he saw fit to nominate trustees who should be incorporated as its governing body, Guy's Hospital assimilated more closely (in its charter, endowment, salaried medical and surgical staff, and its fees for admission) to the chartered institutions than to the voluntary subscription hospitals.

In these particulars Guy's charity stands alone among the hospitals of the eighteenth century, for although many of them owed their origin to the legacies or benefactions of private individuals, with which land was purchased and a building erected and furnished, these were dependent for their maintenance in great measure upon voluntary subscriptions.

The distinction of being the first private citizen to conceive the erection and endowment of a public hospital at his own expense belongs, however, not to Guy, but to Dr. Richard Steevens, of Dublin, who, dying in 1710, bequeathed his real estate, yielding over £600 per annum, in reversion after the death of his sister, to trustees, for the erection and endowment of a hospital near Dublin for the relief and maintenance of curable poor persons. This was incorporated in 1730, and opened to the public three years later; but, like the majority of Irish hospitals, it was maintained in greater part by Parliamentary grants. Similarly John Addenbrooke, M.D., of Cambridge, who died in 1719, left a sum of £4,500, contingent upon the death of his widow, to "erect, or hire, and maintain a small physical hospital in Cambridge for the reception of the sick poor." Delayed by various causes, the design was not carried into execution until 1766, when it was incorporated as "a general hospital to be supported by voluntary subscriptions"; but the publication of Addenbrooke's bequest not improbably supplied Guy with the idea, elaborated under the guidance of the illustrious Mead, of establishing on a scale commensurate with his greater wealth the noble charity bearing his name.

The history of the Irish hospital movement has yet to be written. Initiated by the will of Richard Steevens in 1710, its first development occurred in Cork in 1720, when a proposal was published for the erection by voluntary subscriptions of an infirmary adjoining the Greencoat School in that city, among the contributors to which was the ubiquitous Henry Hoare of the City of London. This building, opened in 1721-2, affords the earliest instance, other than the Westminster Infirmary, of an establishment in the British Isles for the reception and treatment of the sick poor erected and supported by voluntary contributions. The City of Dublin was provided with its earliest charity—the Jervis Street Hospital—in 1728 at the instigation of six surgeons, and was followed in 1734 by Mercer's Hospital, so called after its original benefactress.

Thus to the charity of private individuals Dublin owed the foundation of its three earliest public hospitals—Jervis Street opened in 1728, Steevens' in 1733, and Mercer's in 1734—the first and last of which were maintained entirely by voluntary subscriptions (the other by its endowment), until in 1765 they, like the county infirmaries, became entitled by an Act of the Irish Parliament to an annual municipal grant towards their support.

The solitary instance in the British Isles of a voluntary hospital which was directly due to the initiative of a medical corporation is supplied at Edinburgh, in which city the scheme of supplying the sick poor with the advice and treatment of a physician free of expense had been adopted by its College of Physicians as early as 1682, when that Society had received its charter.

It is recorded that the physicians had often found their efforts unavailing in consequence of the want of suitable accommodation and nursing (there being at that time no workhouse or infirmary for the reception of the sick poor), and in 1725-6 a subscription was raised under the auspices of the College for the provision of a public infirmary, which was opened for the admission of out-patients in 1729. A similar institution was erected at Aberdeen in 1741.

The fourth decade was responsible for the opening of St. George's Hospital near Hyde Park Corner, it being an offshoot from the parent society in the lower part of Westminster, and established, ostensibly for the purpose of supplying the needs of the new suburb arising on the western outskirts of the city, upon a site long famous for its salubrity.

In the absence of State aid or munificence such as that of Steevens, Addenbrooke, and Guy, the voluntary subscription principle afforded the only means of erecting and sustaining public hospitals; and although, with the exceptions of Cork and Edinburgh, it had not as yet (1733-4) found practical application outside Westminster, attempts to raise funds for that purpose had been made at Bath, Northampton, and other places, which from one cause or another had not met with success. All that had occurred in the country districts was the provision of workhouses; and even these were few and far between. The absence of such at Winchester was used as an argument in favour of the erection of an infirmary in that city, and as the first of the county hospitals this merits further notice. Moreover, it is possible to trace step by step in contemporary literature the promotion of the scheme, the publication of the first proposal, the objections raised and confuted, the difficulties met with and surmounted, until the design reached its final consummation.

* Previous articles appeared on December 13, 1913, January 17 and 31, and February 14, 1914.

† The only other institution for incurables dating from the first half of the eighteenth century was established in Dublin in 1744.

